# Supplementary material for: ERBB2 mRNA Expression and Response to Ado-Trastuzumab Emtansine (T-DM1) in HER2-Positive Breast Cancer
Source: Cancers (Basel). 2020 Jul 14;12(7):1902. doi: 10.3390/cancers12071902 (PMC7409149; doi:10.3390/cancers12071902)
Supplement: Supplementary file 1 [file cancers-12-01902-s001.pdf]

cancers

MDPI

Supplementary Materials

ERBB2 mRNA Expression and Response to Ado-Trastuzumab Emtansine (T-DM1) in HER2-Positive Breast Cancer

Table S1. Proportions of *ERBB2*-high tumors across cancer types. *ERBB2* mRNA was evaluated in 10,071 cancers. *ERBB2*-high tumors were identified in 15 cancer-types as shown below, while no *ERBB2*-high tumors were identified in 18 cancer-types, including acute myeloid leukemia (n = 173), adrenocortical carcinoma (n = 78), brain lower grade glioma (n = 514), cholangiocarcinoma (n = 36), diffuse large B-cell lymphoma (n = 48), glioblastoma multiform (n = 160), kidney chromophobe (n = 65), kidney renal clear cell carcinoma (n = 510), kidney renal papillary cell carcinoma (n = 283), liver hepatocellular carcinoma (n = 366), mesothelioma (n = 87), pheochromocytoma and paraganglioma (n = 178), sarcoma (n = 253), skin cutaneous melanoma (n = 443), testicular germ cell tumors (n = 149), thymoma (n = 119), thyroid carcinoma (n = 498), uveal melanoma (n = 80).

Cancer Type	Total (*n*)	ERBB2-low (*n*)	ERBB2-High (*n*)	% ERBB2-High
Breast Invasive Carcinoma HER2 Positive	112	46	66	58.93
Breast Invasive Carcinoma	1082	991	91	8.41
Stomach Adenocarcinoma	412	386	26	6.31
Esophageal Adenocarcinoma	181	170	11	6.08
Cervical Squamous Cell Carcinoma	294	282	12	4.08
Bladder Urothelial Carcinoma	407	391	16	3.93
Rectum Adenocarcinoma	154	148	6	3.90
Pancreatic Adenocarcinoma	177	171	6	3.39
Uterine Corpus Endometrial Carcinoma	527	516	11	2.09
Uterine Carcinosarcoma	57	56	1	1.75
Colon Adenocarcinoma	438	433	5	1.14
Ovarian Serous Cystadenocarcinoma	300	297	3	1.00
Head and Neck Squamous Cell Carcinoma	515	510	5	0.97
Lung Squamous Cell Carcinoma	484	480	4	0.83
Lung Adenocarcinoma	510	507	3	0.59
Breast Invasive Carcinoma HER2 Negative	645	643	2	0.31
Prostate Adenocarcinoma	493	492	1	0.20
ALL	6788	6519	269	3.96

Table S2. Table of abbreviations.

Cancer Type	Abbreviation
Acute Myeloid Leukemia	AML
Adrenocortical Carcinoma	ACC
Bladder Urothelial Carcinoma	BLCA
Brain Lower Grade Glioma	BLGG
Breast Invasive Carcinoma	BRCA
Breast Invasive Carcinoma HER2 Negative	BRCA.HER2-
Breast Invasive Carcinoma HER2 Positive	BRCA.HER2+
Cervical Squamous Cell Carcinoma	CESC
Cholangiocarcinoma	CHOL
Colon Adenocarcinoma	COAD
Diffuse Large B-Cell Lymphoma	DLBCL
Esophageal Adenocarcinoma	EAC
Glioblastoma Multiforme	GBM
Head and Neck Squamous Cell Carcinoma	HNSC
Kidney Chromophobe	KICG
Kidney Renal Clear Cell Carcinoma	KIRC
Kidney Renal Papillary Cell Carcinoma	KIRP
Liver Hepatocellular Carcinoma	LIHC
Lung Adenocarcinoma	LUAD
Lung Squamous Cell Carcinoma	LSCC
Mesothelioma	MESO
Ovarian Serous Cystadenocarcinoma	OVSC
Pancreatic Adenocarcinoma	PAAD
Pheochromocytoma and Paraganglioma	PCPG
Prostate Adenocarcinoma	PRAD
Rectum Adenocarcinoma	REAC
Sarcoma	SARC
Skin Cutaneous Melanoma	SKCM
Stomach Adenocarcinoma	STAD
Testicular Germ Cell Tumors	TGCT
Thymoma	THYM
Thyroid Carcinoma	THCA
Uterine Carcinosarcoma	UCS
Uterine Corpus Endometrial Carcinoma	UCEC
Uveal Melanoma	UVM

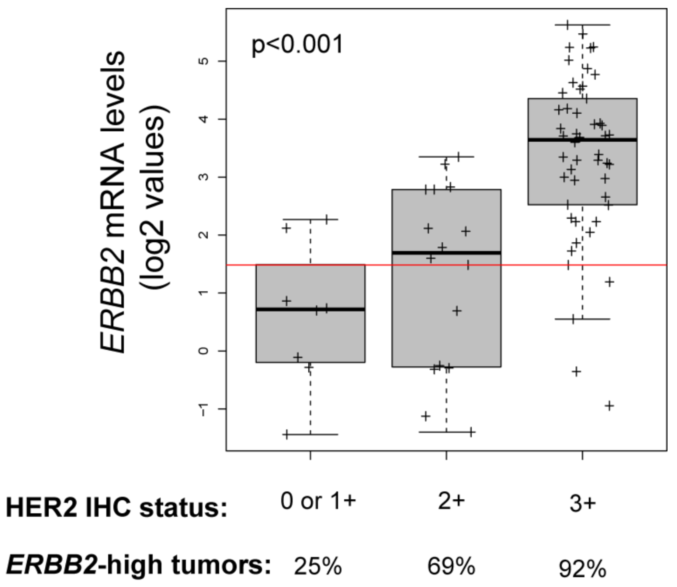

Figure S1. Distribution of *ERBB2* mRNA levels across HER2 IHC subgroups of tumors of the T-DM1 cohort. The proportion of *ERBB2*-high tumors is indicated as defined by the ERBB2 mRNA cutoff (shown as a red line).

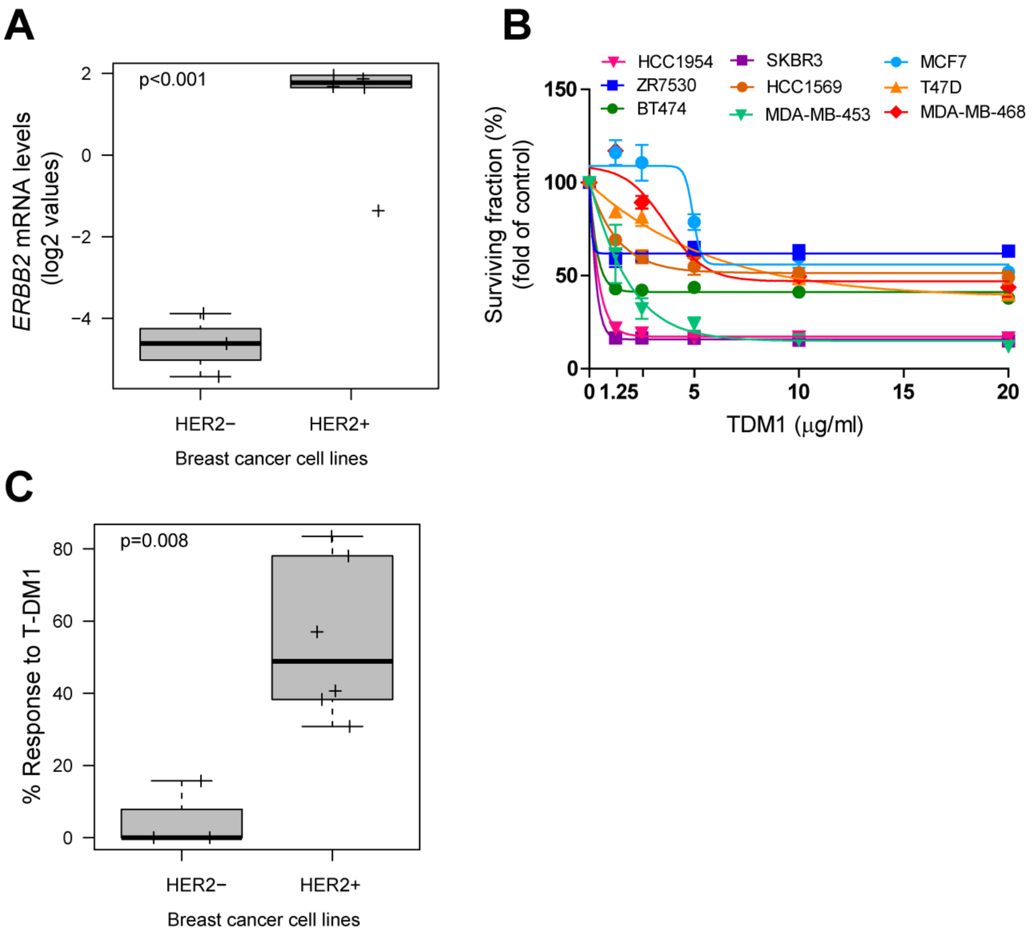

Figure S2. *ERBB2* mRNA levels and T-DM1 response in BC cell lines. (**A**) *ERBB2* mRNA in HER2-negative and HER2+ BC cell lines. (**B**) Surviving fraction (%) of 9 BC cell lines upon 72 h of treatment with increasing concentrations of T-DM1. Data points represent the mean; error bars represent the standard error of the mean of 3 independent experiments. (**C**) BC cell lines response to 1.25 mg/mL T-DM1 expressed as 1-cell viability (%). *p*-values were determined using a two-tailed unpaired t-test.

A

HER2 IHC group	*ERBB2* median (log2 values)	
	HCB	TCGA
0	-1.886334137	12.09052929
1+	-1.58055385	12.59103276
2+/FISH-	-1.283708425	12.86175768
2+/FISH+	-0.046650515	13.71543238
3+	2.712896747	16.54240248

B

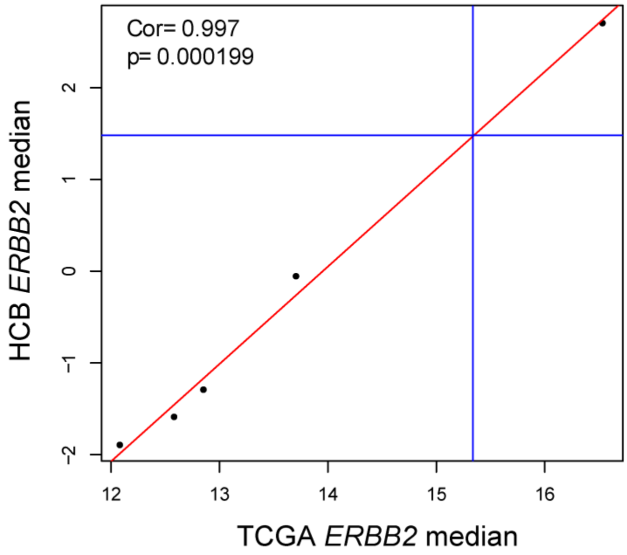

Figure S3. Determination of an *ERBB2* mRNA cutoff for the TCGA dataset. (**A**) *ERBB2* median expression levels across HER2 IHC groups of the primary BC HCB and TCGA datasets. (**B**) Pearson's correlation between the median *ERRB2* mRNA log2 expression values in each HER2 IHC subgroup of the HCB and TCGA datasets.

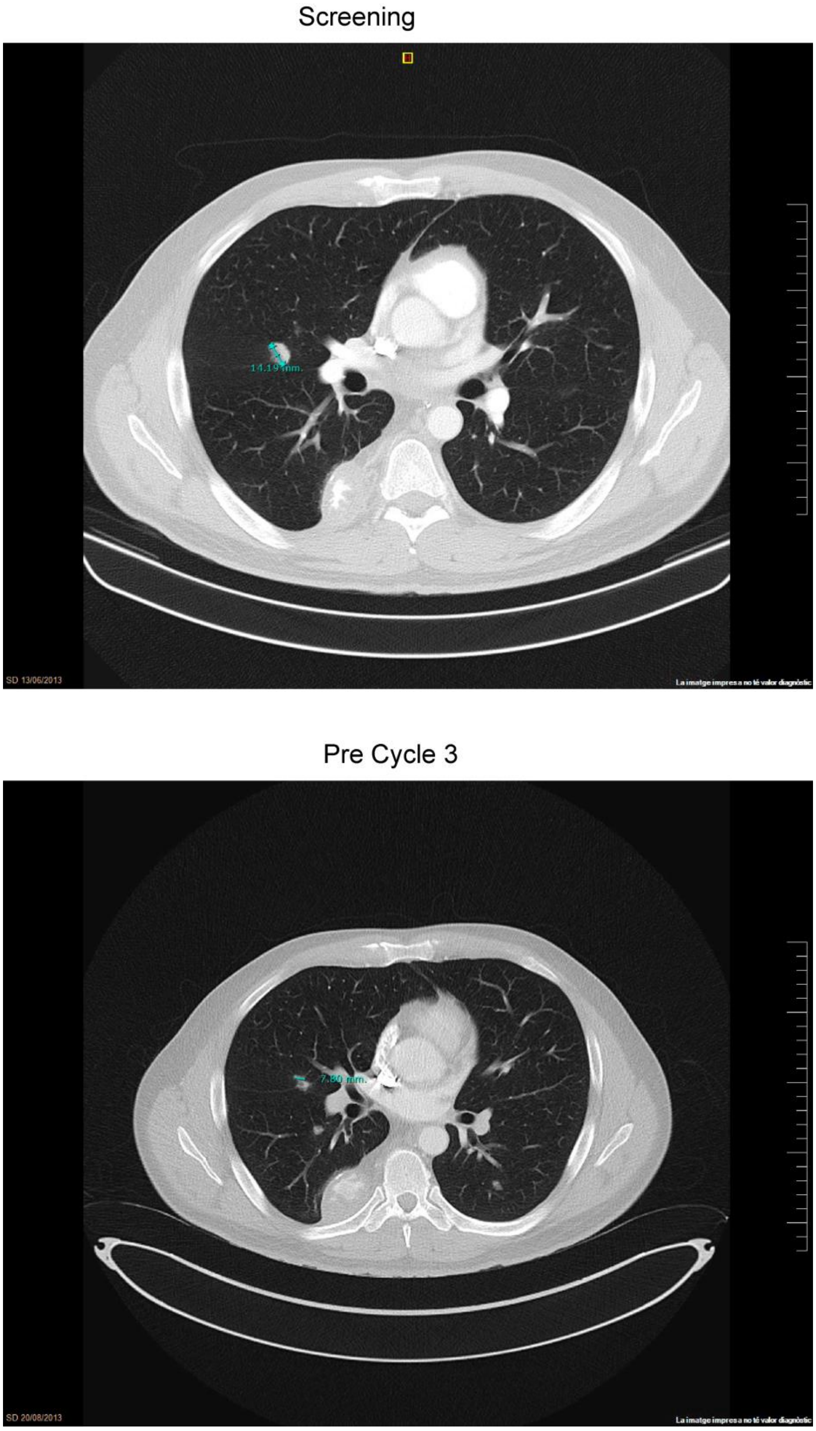

Figure S4. Partial response to T-DM1 in gastric cancer patient. CT scan images of right upper lobe lung metastasis at screening and after 2 complete cycles of T-DM1 treatment. Screening image scale bar: 222 mm. Pre Cycle 3 image scale bar: 245 mm.
